# Temporal Trends and Disparities in Chronic Liver Disease Mortality: An Analysis Using National Center for Health Statistics (NCHS) Data Ranging From 1999 to 2023

**DOI:** 10.7759/cureus.83957

**Published:** 2025-05-12

**Authors:** Victor C Ezeamii, Chukwujindu I Arinzechi, Oghenemaro O Oghotuoma, Uwakmfonabasi Umoudoh, Afolake A Adebayo, Ogechukwu H Nnabude, Taiwo Ajani, Bolaji Ayinde, Chinelo Anyaorah, Gift Ojukwu

**Affiliations:** 1 Public Health, Jiann-Ping Hsu College of Public Health, Georgia Southern University, Statesboro, USA; 2 Internal Medicine, May Pen Hospital, May Pen, JAM; 3 General Internal Medicine, Walsall Healthcare NHS Trust, Walsall, GBR; 4 General Practice, Mersey Care NHS Foundation Trust, Southport, GBR; 5 Family Medicine, Nnamdi Azikiwe University, Nnewi, NGA; 6 General Medicine, Windsor University School of Medicine, Cayon, USA; 7 Internal Medicine, Obafemi Awolowo University, Ile-Ife, NGA; 8 Internal Medicine, Northeast Georgia Medical Center Gainesville, Gainesville, USA; 9 Accident and Emergency, Kingston Hospital, Kingston, JAM; 10 General Practice, Leeds Teaching Hospitals NHS Trust, Leeds, GBR

**Keywords:** chronic liver disease, covid-19, epidemiology, gender disparities, mortality trends, public health, racial disparities

## Abstract

Background: Chronic liver disease (CLD) remains a significant global health concern, with fluctuating mortality trends over the past two decades. This study analyzes age-adjusted CLD mortality rates using data from the Centers for Disease Control and Prevention Wide-Ranging Online Data for Epidemiologic Research (CDC WONDER) from 1999 to 2023, highlighting temporal trends and demographic disparities.

Methods: We conducted a retrospective analysis of CLD mortality rates stratified by year, gender, race, age group, and Hispanicity. Age-adjusted mortality rates were computed using direct standardization, and trends were examined over different periods.

Results: Between 1999 and 2006, CLD mortality rates declined from 9.6 to 8.8 per 100,000 population. However, from 2007 onward, mortality rates increased, peaking at 14.5 in 2021, largely driven by the COVID-19 pandemic. Males exhibited consistently higher mortality rates than females. Racial disparities were evident, with American Indian/Alaska Native populations experiencing the highest mortality rates. Age-related trends showed the greatest burden in older adults, while mortality rates in younger populations also rose in recent years. Hispanic individuals demonstrated a significant increase in CLD mortality, particularly post-2010.

Conclusion: CLD mortality has risen significantly since 2007, with a pronounced peak in 2021. Despite a post-pandemic decline, rates remain above pre-2019 levels. These findings emphasize the significance of customized public health interventions and strategies tackling alcohol use, various metabolic risk factors, and healthcare access, especially for high-risk individuals and populations.

## Introduction

Chronic liver disease (CLD) is a progressive condition characterized by persistent inflammation, fibrosis, and impaired liver function that persists for at least six months, often resulting from various underlying causes, including hepatitis, alcohol abuse, or metabolic disorders [1]. CLD affects the major hepatic processes, such as bile excretion, protein synthesis, and metabolic byproduct detoxification. CLD encompasses a spectrum of diseases, including cirrhosis and hepatocellular carcinoma (HCC), leading to significant morbidity and mortality. Symptoms range from fatigue and jaundice to ascites and hepatic encephalopathy [2]. Diagnosis relies on liver function tests, imaging, and biopsy. Management focuses on addressing the underlying cause, lifestyle modifications, and pharmacological interventions, with liver transplantation as a last resort [3]. Nutritional therapy plays a crucial role in preventing malnutrition and sarcopenia in CLD patients. Early diagnosis and multidisciplinary care are essential to improving patient outcomes and quality of life in individuals with CLD [4].

According to the National Vital Statistics Report 2017 from the Centers for Disease Control and Prevention, approximately 4.5 million adults in the United States (US) had CLD and cirrhosis, accounting for 1.8% of the adult population. In the same year, CLD and cirrhosis caused 41,473 deaths, with a mortality rate of 12.8 per 100,000 population [1]. Globally, the total number of CLD cases, spanning all disease stages, is estimated at 1.5 billion [4]. The most prevalent causes include non-alcoholic fatty liver disease (NAFLD) (59%), hepatitis B virus (HBV) (29%), hepatitis C virus (HCV) (9%), and alcohol-related liver disease (ALD) (2%) [5]. CLD remains a major global health burden, with geographic and socioeconomic disparities influencing its prevalence and outcomes.

The pathogenesis of CLD is a progressive process characterized by persistent hepatic inflammation, fibrosis, and eventual liver dysfunction [1]. The pathophysiology of CLD involves hepatocellular injury triggered by various etiological factors such as HBV, HCV, ALD, NAFLD, and autoimmune or metabolic disorders [5-7]. Repeated hepatocyte injury leads to the cascading of immune responses that include the activation of Kupffer cells alongside the release of pro-inflammatory cytokines, including interleukin (IL)-6, tumor necrosis factor-alpha (TNF-α), and interleukin 1 beta (IL-1β) [6-7]. Such immune signals significantly contribute to the recruitment of inflammatory cells and the subsequent activation of hepatic stellate cells (HSCs) known to play a key role in fibrogenesis through the production of excessive extracellular matrix (ECM) components [5-7]. Such immune-mediated inflammation and the fibrotic responses have been acknowledged to underlie CLD's progressive nature [6-7]. As fibrosis progresses, it disrupts normal liver architecture, leading to portal hypertension, hepatocellular dysfunction, and an increased risk of cirrhosis [8]. Chronic inflammation promotes oxidative stress, mitochondrial dysfunction, and hepatocyte apoptosis, further exacerbating liver damage [9]. In advanced stages, liver cirrhosis develops, increasing the risk of complications such as hepatic decompensation, ascites, hepatic encephalopathy, and HCC. Without timely intervention, CLD ultimately results in end-stage liver disease (ESLD), necessitating liver transplantation [9-10].

The Centers for Disease Control and Prevention Wide-Ranging Online Data for Epidemiologic Research (CDC WONDER) database provides comprehensive mortality data in the US, offering vital statistics on causes of death, demographic trends, and public health patterns [11]. Derived from death certificates, it includes detailed information on CLD mortality. This study utilizes CDC WONDER to analyze age-adjusted death rates, assessing temporal trends, demographic disparities, and risk factors associated with CLD mortality. By identifying high-risk populations, the findings will enhance understanding of disease burden, guide public health policies, and support targeted interventions.

## Materials and methods

Data source and study design

This study is a retrospective observational analysis utilizing mortality data from the CDC WONDER database. CDC WONDER compiles death certificate data from all US states and territories, providing standardized information on mortality trends, including underlying and contributing causes of death. The study focuses on CLD mortality, specifically analyzing age-adjusted death rates from 1999 to 2023. Data were extracted from the CDC WONDER public-use mortality files, ensuring national representativeness and robust epidemiological insights.

Study population and variables

The study population included all reported deaths where CLD was listed as the primary or contributing cause of mortality. Inclusion criteria encompassed cases classified under the International Classification of Diseases, 10th Revision (ICD-10), codes explicitly K70-K77, representing various forms of CLD, including alcoholic liver disease, NAFLD, and viral hepatitis-related cirrhosis. The dataset included demographic variables such as age, sex, race/ethnicity, and geographic region. Since this study relied on secondary mortality data, no direct participant recruitment or questionnaire administration was involved.

Data collection and quality assurance

CDC WONDER aggregates mortality data from state health departments based on information recorded on death certificates completed by physicians, medical examiners, and coroners. The system follows standardized protocols to ensure data accuracy and consistency in cause-of-death reporting. Automated and manual validation processes minimize classification errors and inconsistencies. Age-adjusted mortality rates were computed using the direct standardization method based on the US 2000 standard population, allowing reliable comparisons across demographic groups and periods.

Variables of interest

The primary outcome variable in this study was the age-adjusted death rate for CLD, expressed per 100,000 population. Key demographic variables included age groups (<25, 25-44, 45-64, and ≥65 years), sex (male and female), and race/ethnicity (White individuals, Black individuals, Hispanic individuals, Asian/Pacific Islander individuals, Native American individuals, and other racial groups). These variables comprehensively assessed CLD mortality trends and their variation across different demographic subgroups.

Data analysis and statistical methods

Statistical analyses were conducted using IBM SPSS Statistics for Windows, Version 30 (Released 2024; IBM Corp., Armonk, New York, USA). Descriptive statistics were used to summarize mortality trends, and age-adjusted death rates were calculated through direct standardization. Trends over time were analyzed using Joinpoint regression models to detect significant shifts in mortality rates. Differences between groups (age, gender, race/ethnicity) were evaluated using chi-square tests for categorical variables and t-tests or ANOVA for continuous variables. A significance level of a p-value less than 0.05 was applied for all statistical tests.

Ethical considerations

This study utilized publicly available, de-identified mortality data from CDC WONDER, which does not require direct patient consent or Institutional Review Board (IRB) approval. However, the US Department of Health and Human Services (HHS) guidelines upheld ethical principles of data privacy and confidentiality. No personal identifiers were included in the dataset, ensuring compliance with the Health Insurance Portability and Accountability Act (HIPAA).

## Results

Table 1 provides a detailed breakdown of age-adjusted incidence rates (per 100,000) with 95% CI for CLD across different racial and age groups, stratified by sex and overall population. The statistical significance using ANOVA of CLD-related mortality differences across various demographic groups indicates a statistically significant difference in mortality rates between groups (p < 0.05).

**Table 1:** Age-adjusted incidence rates of CLD by race, age groups, and sex (1999-2023) The values in parentheses represent the 95% confidence interval (CI).

Year	1999	2000	2001	2002	2003	2004	2005	2006	2007	2008	2009	2010	2011	2012	2013	2014	2015	2016	2017	2018	2019	2020	2021	2022	2023	p-value
Deaths	26259	26552	27035	27257	27503	27013	27530	27555	29165	29963	30558	31903	33642	34979	36427	38170	40326	40545	41743	42838	44358	51642	56585	54803	52222	
Population	279040168	281421906	284968955	287625193	290107933	292805298	295516599	298379912	301231207	304093966	306771529	308745538	311591917	313914040	316128839	318857056	321418820	323127513	325719178	327167434	328239523	329484123	331893745	333287557	334914895	
Crude rate	9.4	9.4	9.5	9.5	9.5	9.2	9.3	9.2	9.7	9.9	10	10.3	10.8	11.1	11.5	12	12.5	12.5	12.8	13.1	13.5	15.7	17	16.4	15.6	
Age-adjusted rate	9.6 (9.4-9.7)	9.5 (9.4-9.7)	9.5 (9.4-9.6)	9.4 (9.3-9.5)	9.3 (9.2-9.4)	9.0 (8.8-9.1)	8.9 (8.8-9.1)	8.8 (8.7-8.9)	9.1 (9.0-9.2)	9.2 (9.1-9.3)	9.1 (9.0-9.2)	9.4 (9.3-9.5)	9.7 (9.6-9.8)	9.9 (9.8-10.0)	10.2 (10.0-10.3)	10.4 (10.3-10.5)	10.8 (10.7-11.0)	10.7 (10.6-10.8)	10.9 (10.8-11.0)	11.1 (10.9-11.2)	11.3 (11.2-11.4)	13.3 (13.1-13.4)	14.5 (14.3-14.6)	13.8 (13.7-14.0)	13.0 (12.9-13.1)	
Based on gender																										
Female	6.1 (6.0-6.2)	6.2 (6.0-6.3)	6.3 (6.1-6.4)	6.3 (6.2-6.4)	6.0 (5.9-6.1)	5.9 (5.7-6.0)	5.8 (5.7-5.9)	5.8 (5.7-5.9)	5.9 (5.8-6.0)	6.0 (5.8-6.1)	6.1 (6.0-6.2)	6.2 (6.1-6.3)	6.6 (6.5-6.7)	6.7 (6.6-6.8)	6.8 (6.6-6.9)	7.1 (7.0-7.2)	7.6 (7.4-7.7)	7.5 (7.4-7.6)	7.6 (7.5-7.8)	7.7 (7.6-7.8)	8.0 (7.8-8.1)	9.4 (9.3-9.6)	10.3 (10.2-10.5)	10.0 (9.8-10.1)	9.5 (9.3-9.6)	<0.05
Male	13.5 (13.3-13.8)	13.4 (13.2-13.6)	13.2 (13.0-13.4)	12.9 (12.7-13.1)	13.0 (12.8-13.2)	12.4 (12.3-12.6)	12.4 (12.2-12.6)	12.1 (11.9-12.3)	12.7 (12.5-12.8)	12.7 (12.5-12.9)	12.5 (12.3-12.7)	12.9 (12.7-13.1)	13.1 (12.9-13.3)	13.4 (13.3-13.6)	13.8 (13.7-14.0)	14.1 (13.9-14.2)	14.5 (14.3-14.7)	14.3 (14.2-14.5)	14.5 (14.3-14.7)	14.7 (14.6-14.9)	15.1 (14.9-15.3)	17.5 (17.3-17.7)	18.9 (18.7-19.1)	18.0 (17.8-18.2)	16.8 (16.7-17.0)	
Based on race																										
American Indian or Alaska Native	24.8 (22.5-27.0)	24.3 (22.1-26.5)	22.7 (20.7-24.8)	22.8 (20.8-24.8)	22.3 (20.4-24.2)	22.3 (20.3-24.2)	21.6 (19.8-23.4)	20.9 (19.1-22.7)	23.1 (21.3-24.9)	23.6 (21.8-25.4)	21.3 (19.7-23.0)	22.8 (21.1-24.5)	22.9 (21.3-24.6)	25.3 (23.6-27.0)	24.8 (23.2-26.5)	24.2 (22.7-25.8)	26.4 (24.8-28.0)	26.7 (25.1-28.4)	26.1 (24.5-27.7)	27.7 (26.1-29.3)	27.5 (25.9-29.1)	35.5 (33.7-37.3)	48.2 (46.0-50.3)	40.3 (38.4-42.3)	33.4 (31.6-35.1)	<0.05
Asian or Pacific Islander	3.7 (3.2-4.1)	3.5 (3.1-3.9)	3.5 (3.1-3.9)	3.2 (2.9-3.6)	3.0 (2.7-3.4)	3.2 (2.9-3.6)	3.6 (3.2-4.0)	3.5 (3.2-3.9)	3.3 (2.9-3.6)	3.4 (3.1-3.7)	3.3 (3.0-3.6)	3.2 (2.9-3.6)	3.3 (3.0-3.6)	3.3 (3.0-3.6)	3.3 (3.0-3.6)	3.5 (3.2-3.7)	3.3 (3.0-3.5)	3.4 (3.1-3.7)	3.7 (3.4-3.9)	3.7 (3.4-3.9)	3.7 (3.4-3.9)	4.3 (4.0-4.5)	4.2 (3.9-4.5)	4.1 (3.8-4.4)	4.1 (3.8-4.4)	
Black or African American	10.1 (9.7-10.5)	9.4 (9.1-9.8)	9.3 (8.9-9.6)	8.4 (8.1-8.8)	8.3 (8.0-8.6)	7.8 (7.5-8.1)	7.6 (7.3-7.9)	6.8 (6.5-7.1)	7.2 (6.9-7.5)	6.8 (6.5-7.1)	6.8 (6.5-7.0)	6.7 (6.5-7.0)	7.0 (6.7-7.2)	6.9 (6.6-7.1)	7.3 (7.0-7.5)	7.2 (6.9-7.4)	7.4 (7.1-7.6)	7.1 (6.9-7.4)	7.3 (7.0-7.5)	7.1 (6.9-7.3)	7.3 (7.1-7.6)	8.7 (8.4-9.0)	9.6 (9.3-9.9)	8.6 (8.3-8.9)	8.1 (7.8-8.3)	
White	9.6 (9.4-9.7)	9.6 (9.5-9.8)	9.6 (9.5-9.7)	9.6 (9.5-9.7)	9.6 (9.4-9.7)	9.2 (9.1-9.3)	9.2 (9.1-9.3)	9.1 (9.0-9.2)	9.5 (9.3-9.6)	9.6 (9.5-9.7)	9.6 (9.5-9.7)	9.9 (9.8-10.0)	10.2 (10.1-10.4)	10.5 (10.4-10.7)	10.7 (10.6-10.9)	11.2 (11.0-11.3)	11.7 (11.5-11.8)	11.6 (11.4-11.7)	11.7 (11.6-11.8)	11.9 (11.8-12.0)	12.3 (12.1-12.4)	14.3 (14.2-14.5)	15.6 (15.5-15.8)	14.6 (14.4-14.7)	13.3 (13.2-13.5)	
Based on age groups																										
15-24 years	0 (0-0.1)	0.1 (0.1-0.1)	0.1 (0-0.1)	0.1 (0.1-0.1)	0.1 (0-0.1)	0.1 (0-0.1)	0.1 (0-0.1)	0.1 (0-0.1)	0.1 (0-0.1)	0.1 (0-0.1)	0.1 (0-0.1)	0.1 (0.1-0.1)	0.1 (0.1-0.1)	0.1 (0-0.1)	0.1 (0-0.1)	0.1 (0-0.1)	0.1 (0-0.1)	0.1 (0.1-0.1)	0.1 (0-0.1)	0.1 (0.1-0.1)	0.1 (0.1-0.1)	0.1 (0.1-0.1)	0.1 (0.1-0.2)	0.1 (0.1-0.2)	0.1 (0.1-0.2)	<0.05
25-34 years	1 (0.9-1.1)	1 (0.9-1.1)	1 (0.9-1.1)	1 (0.9-1)	0.9 (0.8-1)	0.8 (0.7-0.9)	0.8 (0.7-0.9)	0.8 (0.7-0.9)	1 (0.9-1.1)	1.1 (1-1.2)	1.1 (1-1.2)	1.2 (1.1-1.3)	1.2 (1.1-1.3)	1.4 (1.3-1.5)	1.6 (1.5-1.7)	1.7 (1.5-1.8)	1.9 (1.8-2)	2.1 (1.9-2.2)	2 (1.9-2.2)	2.2 (2.1-2.3)	2.4 (2.3-2.6)	3.5 (3.4-3.7)	4 (3.8-4.2)	3.9 (3.7-4.1)	3.6 (3.4-3.7)	
35-44 years	7.3 (7.1-7.6)	7.5 (7.2-7.7)	7.4 (7.2-7.7)	7.1 (6.8-7.3)	6.8 (6.6-7.1)	6.4 (6.2-6.6)	6.2 (5.9-6.4)	5.9 (5.7-6.1)	6 (5.8-6.2)	6.1 (5.8-6.3)	6 (5.7-6.2)	5.9 (5.7-6.1)	6 (5.8-6.3)	6.1 (5.9-6.3)	6.2 (5.9-6.4)	6.4 (6.1-6.6)	7 (6.8-7.3)	7 (6.8-7.3)	7.3 (7.1-7.6)	7.5 (7.3-7.8)	8.2 (7.9-8.5)	11.7 (11.4-12)	13.4 (13.1-13.8)	12.6 (12.3-12.9)	11.3 (11-11.6)	
45-54 years	17.4 (17-17.8)	17.7 (17.2-18.1)	18.4 (18-18.9)	18 (17.6-18.5)	18.3 (17.9-18.7)	18 (17.6-18.4)	17.7 (17.3-18.1)	17.8 (17.4-18.2)	18.7 (18.3-19.1)	18.5 (18.1-18.9)	18.7 (18.3-19.1)	19.2 (18.8-19.6)	19.8 (19.4-20.2)	20.1 (19.6-20.5)	20.1 (19.7-20.5)	19.9 (19.4-20.3)	20.5 (20.1-21)	19.5 (19.1-20)	19.6 (19.2-20)	19.6 (19.2-20)	19.8 (19.4-20.2)	23.5 (23.1-24)	25.8 (25.3-26.3)	23.3 (22.8-23.7)	21.9 (21.4-22.4)	
55-64 years	23.7 (23.1-24.3)	23.8 (23.2-24.4)	22.9 (22.3-23.5)	22.8 (22.3-23.4)	22.9 (22.4-23.5)	22.4 (21.9-23)	23.3 (22.7-23.8)	22.6 (22.1-23.1)	24.2 (23.6-24.7)	25 (24.4-25.5)	25.9 (25.3-26.4)	26.8 (26.2-27.3)	28.2 (27.7-28.8)	29.1 (28.6-29.6)	30.4 (29.9-30.9)	31.9 (31.4-32.5)	32.5 (31.9-33)	32.4 (31.9-33)	32.7 (32.2-33.3)	33 (32.4-33.5)	33.9 (33.3-34.4)	38.1 (37.5-38.7)	41.3 (40.7-41.9)	39.2 (38.6-39.8)	35.3 (34.7-35.8)	
65-74 years	30.6 (29.8-31.4)	29.8 (29-30.6)	29.8 (29.1-30.6)	29.3 (28.5-30)	29.2 (28.4-30)	27.4 (26.7-28.2)	26.8 (26.1-27.6)	25.6 (24.9-26.3)	26.2 (25.5-26.9)	26.3 (25.6-27)	25.4 (24.7-26)	26.3 (25.7-27)	26.3 (25.6-26.9)	27.6 (26.9-28.2)	28.1 (27.4-28.8)	29.6 (28.9-30.2)	30.5 (29.9-31.2)	30.7 (30.1-31.4)	31.7 (31-32.3)	32.5 (31.9-33.1)	33.1 (32.4-33.7)	36.5 (35.9-37.2)	38.4 (37.8-39.1)	39 (38.3-39.7)	37.5 (36.9-38.2)	
75-84 years	31.9 (30.9-32.9)	31 (30-32)	30.2 (29.2-31.1)	31.3 (30.4-32.3)	29.9 (29-30.9)	28.7 (27.8-29.7)	28.9 (28-29.8)	28.9 (27.9-29.8)	28.2 (27.3-29.1)	28 (27.1-28.9)	27.2 (26.3-28.1)	27.7 (26.8-28.6)	29.3 (28.4-30.2)	29.3 (28.4-30.3)	29.9 (28.9-30.8)	30.4 (29.5-31.3)	31.9 (31-32.9)	31.9 (31-32.9)	31.3 (30.4-32.3)	32.5 (31.6-33.4)	32.2 (31.3-33.1)	34.5 (33.6-35.4)	36.4 (35.5-37.3)	36.7 (35.8-37.6)	37 (36.2-37.9)	
85+ years	23.2 (21.8-24.7)	23.1 (21.6-24.5)	22.7 (21.3-24.1)	22.5 (21.1-24)	21.2 (19.8-22.5)	21.1 (19.7-22.4)	21.3 (20-22.7)	21.1 (19.8-22.4)	21.7 (20.4-23)	21.9 (20.6-23.2)	21.1 (19.9-22.4)	21.8 (20.5-23)	22.1 (20.9-23.3)	21.4 (20.2-22.6)	23 (21.8-24.2)	23.4 (22.2-24.7)	25.1 (23.8-26.3)	24.5 (23.3-25.7)	26.8 (25.6-28.1)	25.5 (24.2-26.7)	26.5 (25.3-27.8)	27 (25.7-28.2)	31 (29.6-32.4)	30.2 (28.9-31.5)	33.5 (32.1-35)	
Based on Hispanic race																										
Hispanic or Latino	16.1 (15.4-16.7)	16.5 (15.9-17.1)	15.9 (15.3-16.5)	15.7 (15.1-16.2)	14.9 (14.3-15.4)	14.1 (13.6-14.6)	14.1 (13.6-14.6)	13.6 (13.2-14.1)	14.0 (13.6-14.5)	14.0 (13.5-14.5)	14.0 (13.6-14.5)	13.7 (13.2-14.1)	14.1 (13.7-14.6)	14.1 (13.7-14.5)	14.0 (13.6-14.4)	14.5 (14.1-14.9)	14.9 (14.5-15.3)	14.7 (14.3-15.0)	14.3 (13.9-14.6)	14.5 (14.2-14.9)	14.6 (14.2-14.9)	16.4 (16.0-16.7)	17.3 (16.9-17.7)	17.0 (16.6-17.4)	15.9 (15.5-16.2)	0.05
Not Hispanic or Latino	16.1 (15.4-16.7)	16.5 (15.9-17.1)	15.9 (15.3-16.5)	15.7 (15.1-16.2)	14.9 (14.3-15.4)	14.1 (13.6-14.6)	14.1 (13.6-14.6)	13.6 (13.2-14.1)	14.0 (13.6-14.5)	14.0 (13.5-14.5)	14.0 (13.6-14.5)	13.7 (13.2-14.1)	14.1 (13.7-14.6)	14.1 (13.7-14.5)	14.0 (13.6-14.4)	14.5 (14.1-14.9)	14.9 (14.5-15.3)	14.7 (14.3-15.0)	14.3 (13.9-14.6)	14.5 (14.2-14.9)	14.6 (14.2-14.9)	16.4 (16.0-16.7)	17.3 (16.9-17.7)	17.0 (16.6-17.4)	15.9 (15.5-16.2)	

Initially, a decline in mortality rates was observed, with the rate decreasing from 9.6 (95% CI: 9.4-9.7) in 1999 to 8.8 (95% CI: 8.7-8.9) in 2006, suggesting improvements in disease management and public health interventions. However, from 2007 onwards, a reversal in trend was noted, with mortality rates steadily increasing. Between 2007 and 2015, the rate rose from 9.1 (95% CI: 9.0-9.2) to 10.8 (95% CI: 10.7-11.0), reflecting a worsening burden of CLD. The upward trend continued, reaching 11.3 (95% CI: 11.2-11.4) in 2019. A sharp spike was recorded in 2020, with mortality rising to 13.3 (95% CI: 13.1-13.4), likely due to the COVID-19 pandemic. The peak occurred in 2021 at 14.5 (95% CI: 14.3-14.6), followed by a decline to 13.0 (95% CI: 12.9-13.1) in 2023, indicating partial recovery but persistent post-pandemic effects. The age-adjusted incidence rates of CLD by race, age groups, and sex (1999-2023) have been indicated in Table 1.

Based on gender

**Figure 1:**
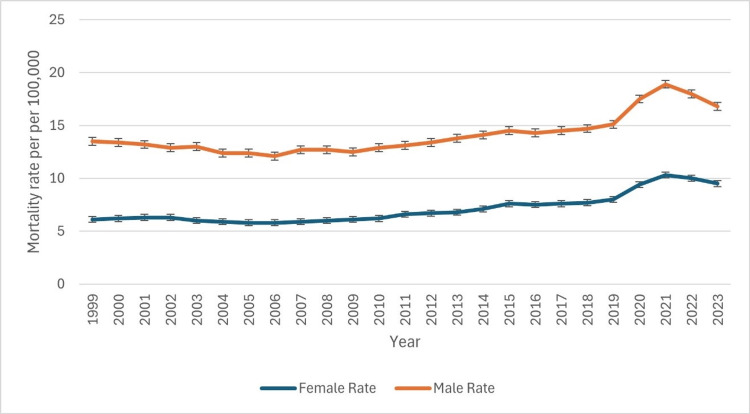
Patterns of underlying causes of death due to chronic liver disease based on gender

The age-adjusted death rates for CLD consistently showed a gender disparity from 1999 to 2023, with higher mortality rates in males than females (Figure 1). In 1999, the male death rate was 13.5 (95% CI: 13.3-13.8), whereas the female rate was significantly lower at 6.1 (95% CI: 6.0-6.2). This male predominance persisted throughout the study period. Between 1999 and 2010, both genders exhibited relative stability, with male rates fluctuating between 12.9 and 13.7 and female rates between 5.8 and 6.3. However, a gradual increase began in 2011, with male rates reaching 14.5 (95% CI: 14.3-14.7) and female rates rising to 7.6 (95% CI: 7.4-7.7) by 2015. The most significant surge occurred from 2019 to 2021, where male mortality spiked from 15.1 (95% CI: 14.9-15.3) to 18.9 (95% CI: 18.7-19.1), while female rates increased from 8.0 (95% CI: 7.8-8.1) to 10.3 (95% CI: 10.2-10.5). This rise may be linked to external factors such as disease prevalence, healthcare access, or lifestyle changes. Post-2021, a slight decline was noted, with male rates dropping to 16.8 (95% CI: 16.7-17.0) and female rates to 9.5 (95% CI: 9.3-9.6) by 2023. Despite this decrease, rates remain higher than pre-2015 levels, emphasizing the persistent gender disparity in CLD mortality. The patterns of underlying causes of death due to CLD based on gender have been indicated in Figure 1 below.

Based on race

**Figure 2:**
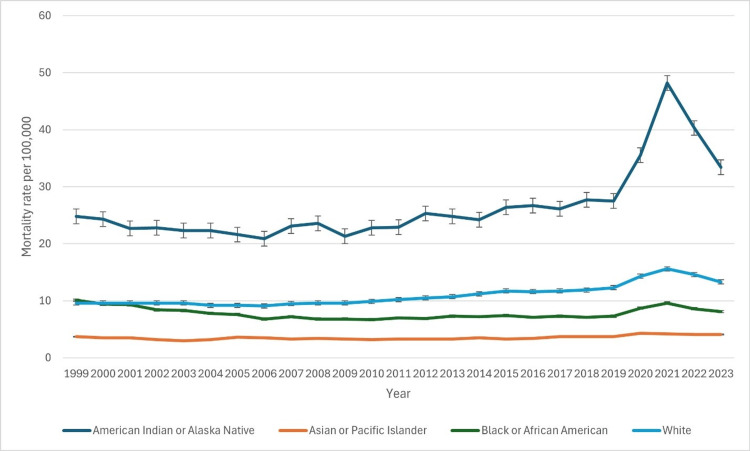
Patterns of underlying causes of death due to chronic liver disease based on race

The age-adjusted mortality rates for CLD varied significantly across racial groups from 1999 to 2023, with notable disparities in trends (Figure 2). The American Indian or Alaska Native (AI/AN) population consistently exhibited the highest mortality rates. In 1999, the age-adjusted rate was 24.8 (95% CI: 22.5-27.0), nearly doubling by 2021 to 48.2 (95% CI: 46.0-50.3). Although rates declined in 2022 (40.3, 95% CI: 38.4-42.3) and 2023 (33.4, 95% CI: 31.6-35.1), they remained significantly elevated compared to other racial groups. Among White individuals, mortality rates increased steadily over the study period. From 9.6 (95% CI: 9.4-9.7) in 1999, rates peaked at 15.6 (95% CI: 15.5-15.8) in 2021. A slight decline followed, with rates at 15.1 (95% CI: 15.0-15.3) in 2022 and 14.3 (95% CI: 14.2-14.4) in 2023. The Black population experienced moderate fluctuations. In 1999, the rate was 10.1 (95% CI: 9.7-10.5), decreasing to 6.8 (95% CI: 6.5-7.0) by 2009. However, after 2010, rates rose again, peaking at 10.2 (95% CI: 9.6-9.9) in 2021 before declining to 8.1 (95% CI: 7.8-8.3) in 2023. The Asian or Pacific Islander population consistently had the lowest mortality rates, starting at 3.7 (95% CI: 3.2-4.1) in 1999 and peaking at 4.4 (95% CI: 4.0-4.5) in 2020 and 2022. By 2023, the rate slightly declined to 4.1 (95% CI: 3.8-4.4). Data for Native Hawaiian or Other Pacific Islander and multiracial populations became available in 2021. The Native Hawaiian or Other Pacific Islander group had a rate of 8.5 (95% CI: 6.5-10.9) in 2021, declining to 7.2 (95% CI: 5.4-9.4) in 2023. The multiracial group had a rate of 8.0 (95% CI: 7.2-8.8) in 2021, decreasing to 7.2 (95% CI: 6.5-7.9) in 2023. The patterns of underlying causes of death due to CLD based on race have been indicated in Figure 2 below.

Based on age groups

**Figure 3:**
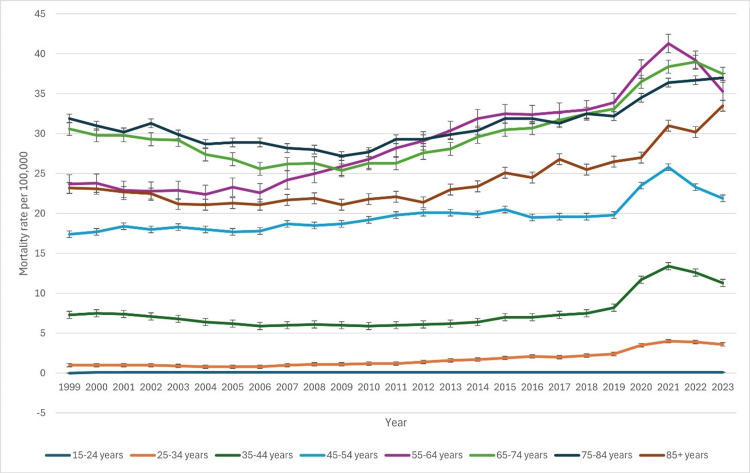
Patterns of underlying causes of death due to chronic liver disease based on age groups

Mortality rates varied significantly across different age groups over time, with older populations experiencing higher rates (Figure 3). In 1999, the lowest mortality rate was observed in the 15-24 years age group at 0 (95% CI: 0-0.1) per 100,000, while the highest was in the 75-84 years group at 31.9 (95% CI: 30.9-32.9). The 85+ years group had a mortality rate of 23.2 (95% CI: 21.8-24.7), slightly lower than the 75-84 years group. From 1999 to 2016, younger age groups (15-24 and 25-34 years) maintained relatively low mortality rates, with minor increases in later years. For instance, in 1999, the mortality rate for the 25-34 years group was 1.0 (95% CI: 0.9-1.1), which increased to 2.1 (95% CI: 1.9-2.2) in 2016. The 35-44 years group remained relatively stable, with a slight decrease from 7.3 (95% CI: 7.1-7.6) in 1999 to 7.0 (95% CI: 6.8-7.3) in 2016.

Conversely, older age groups showed increasing mortality trends. The 45-54 years group experienced a rise from 17.4 (95% CI: 17.0-17.8) in 1999 to 19.5 (95% CI: 19.1-20.0) in 2016. A similar upward trajectory was observed in the 55-64 years group, where rates climbed from 23.7 (95% CI: 23.1-24.3) to 32.4 (95% CI: 31.8-33.0) over the same period. The 65-74 years group exhibited a notable increase, from 30.6 (95% CI: 29.8-31.4) in 1999 to 32.3 (95% CI: 31.7-32.9) in 2016. The mortality rate in the 75-84 years category remained consistently high, peaking at 31.9 (95% CI: 31.0-32.9) in 2015. In the 85+ years group, rates showed a steady increase from 23.2 (95% CI: 21.8-24.7) in 1999 to 26.3 (95% CI: 25.7-26.9) in 2016, reflecting an overall upward trend in mortality among the elderly. The patterns of underlying causes of death due to CLD based on age groups have been indicated in Figure 3 below.

Based on Hispanic origin

**Figure 4:**
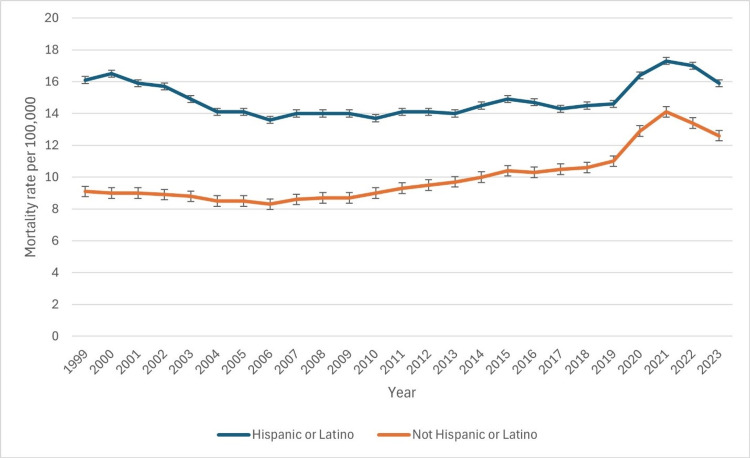
Patterns of underlying causes of death due to chronic liver disease based on Hispanic race

From 1999 to 2023, mortality rates among the Hispanic population exhibited an overall increasing trend (Figure 4). In 1999, the crude death rate was 8.8 per 100,000, with an adjusted rate of 16.1 (95% CI: 15.4-16.7). The rate remained relatively stable over the next decade, with slight declines in 2003 (14.9) and 2006 (13.6). However, after 2010, a consistent upward trend emerged, with the crude rate rising from 8.6 in 2010 to 10.6 in 2015 and 11.4 in 2019. A sharp increase occurred in 2020, with the crude rate reaching 13.1 and an adjusted rate of 16.4 (16.0-16.7), likely influenced by the COVID-19 pandemic. The peak was observed in 2021 at 14.1 per 100,000 (adjusted rate: 17.3). A slight decline followed in 2022 (17.0) and 2023 (15.9), suggesting potential stabilization. These findings highlight a rising mortality burden among Hispanic individuals, exacerbated by public health crises. The patterns of underlying causes of death due to CLD based on Hispanic race have been indicated in Figure 4 below.

## Discussion

The findings reveal distinct temporal fluctuations, significant gender disparities, racial variations, and age-related mortality patterns. Furthermore, the impact of external factors, particularly the COVID-19 pandemic, underscores the need for targeted interventions to address CLD-related mortality.

Our results indicate that CLD mortality initially declined between 1999 and 2006, followed by a reversal in trends from 2007 onwards. The period from 1999 to 2006 saw a gradual decline in mortality from 9.6 per 100,000 in 1999 to 8.8 per 100,000 in 2006, indicating possible improvements associated with healthcare access, early disease detection, and advancements in CLD management [12-13]. However, after 2007, a steady increase was observed, peaking at 14.5 per 100,000 in 2021, likely influenced by increased alcohol consumption, metabolic risk factors, and the impact of the COVID-19 pandemic [14].

The sharp increase in 2020 and 2021, coinciding with the COVID-19 pandemic, suggests multiple contributory factors. Several studies have indicated that the pandemic exacerbated alcohol consumption, led to healthcare disruptions, and heightened the severity of liver disease among infected patients [15]. While post-pandemic recovery was observed in 2023 (13.0 per 100,000), mortality rates remain above pre-2019 levels, necessitating continued surveillance and policy interventions.

Consistent gender disparities in relation to CLD mortality, including the higher mortality rates in males compared to females, have been disclosed throughout the study period, with a sharper increment in the mortality rates of both genders being noted in 2019 and 2021. The observed disparities might be influenced by various biological factors, including differences in hormonal regulations and alcohol metabolism, alongside sociocultural factors that include higher consumption of alcohol among males and variations in healthcare-seeking behaviors [16]. Further aspects, including occupational exposure to various liver toxins and unequal access to preventive healthcare services, might additionally contribute to the disparities [16]. Additionally, men are more likely to engage in high-risk alcohol consumption, which remains a key driver of CLD progression [17]. Despite a decline in mortality post-2021, male rates in 2023 (16.8) remain substantially higher than female rates (9.5), indicating the need for targeted gender-specific interventions, including alcohol reduction programs and early screening strategies.

The study also identified stark racial disparities in CLD mortality. AI/AN populations experienced the highest mortality rates, rising from 24.8 per 100,000 in 1999 to a peak of 48.2 in 2021, before declining to 33.4 in 2023. The disproportionately high burden among AI/AN populations aligns with previous literature attributing this disparity to socioeconomic determinants, higher alcohol use, and limited access to specialized healthcare services [18]. Similarly, White individuals exhibited a consistent increase in CLD mortality, peaking at 15.6 in 2021, reflecting trends in alcohol-associated liver disease and obesity-related NAFLD [19]. In contrast, Black populations experienced a decline in mortality from 10.1 in 1999 to 6.8 in 2009, followed by an upward trend post-2010, peaking at 10.2 in 2021. This fluctuation may be related to improvements in healthcare access in earlier years, followed by the worsening obesity epidemic and disparities in NAFLD diagnosis and management [20].

The lowest mortality rates were observed in Asian or Pacific Islander populations, remaining relatively stable over time, consistent with lower alcohol consumption rates and genetic predisposition to alcohol intolerance [21]. The emergence of mortality data for Native Hawaiian, Other Pacific Islander, and multiracial populations after 2021 highlights the need for further studies to understand these trends better.

Mortality rates varied considerably across age groups, with older individuals exhibiting the highest burden. The 75-84 years group consistently had the highest mortality, peaking at 31.9 in 2015, while the 85+ years group saw a steady increase over time, reaching 26.3 in 2016. This pattern is consistent with the natural history of liver disease, where older adults are more likely to develop decompensated cirrhosis and HCC [22].

Conversely, younger age groups (25-34 years and 35-44 years) demonstrated relatively low mortality, though recent trends indicate an increase in CLD-related deaths among them. The rise from 1.0 in 1999 to 2.1 in 2016 for the 25-34 group suggests a concerning trend in early-onset liver disease, potentially driven by increased alcohol use disorder and obesity [23]. Addressing these trends through early intervention and public health initiatives targeting younger demographics is imperative.

The persistent increase in CLD mortality, particularly post-2007, necessitates urgent public health action. Policies targeting alcohol use reduction, obesity management, and enhanced access to liver disease screening and treatment must be prioritized. The racial and gender disparities highlight the need for culturally tailored interventions, particularly for high-risk groups such as AI/AN populations and middle-aged males.

Future research should explore the impact of novel therapeutic interventions, the role of social determinants of health in CLD progression, and the long-term consequences of the COVID-19 pandemic on liver disease outcomes. Additionally, longitudinal studies evaluating the effectiveness of national public health campaigns in mitigating CLD mortality are warranted.

Strengths and limitations

A major strength of this study is its use of a large, nationally representative dataset from the National Center for Health Statistics, ensuring robust and generalizable findings. The extended study period (1999-2023) allows for a thorough analysis of mortality trends and key shifts in disease burden. Stratification by demographic factors offers crucial insights into disparities, aiding targeted public health efforts. The study also assesses the impact of external factors like COVID-19 on CLD mortality. However, limitations exist. The observational nature limits causal inferences, and reliance on death certificates may introduce misclassification bias. Socioeconomic and lifestyle factors were not directly analyzed. Additionally, various comorbidities with the potential to confound CLD mortality have not been directly included in this study, given that the study mainly focused on mortality data drawn from the National Center for Health Statistics, which mainly depends on death certificates. Future research incorporating patient-level clinical data is essential for more targeted interventions to reduce CLD mortality.

## Conclusions

In conclusion, this study has provided a broader analysis of the CLD mortality trends across the US, indicating notable temporal fluctuations, age-associated mortality patterns, racial variations, and significant gender disparities. The findings have disclosed a decline in CLD mortality rates between 1999 and 2006, followed by a consistent increment from 2007 onwards, with a sharper surge between 2020 and 2021, which is a period that coincides with the COVID-19 pandemic. Further, males constantly exhibited higher mortality rates in comparison to females, even as the American Indian/Alaska Native populations were found to have experienced the highest burden of mortality, highlighting the requirement for targeted interventions. Regarding age, older adults had the highest mortality rate, even as the younger persons have exhibited a concerning increment in recent times. Such disparities and variations underscore the significance of customized public health interventions and strategies tackling alcohol use, various metabolic risk factors, and healthcare access, especially for high-risk individuals and populations. Prospective efforts should emphasize the expansion of early screening, improvement of access to healthcare, and development of culture-sensitive interventions to mitigate the CLD mortality burden.
